# Historical comparisons reveal multiple drivers of decadal change of an ecosystem engineer at the range edge

**DOI:** 10.1002/ece3.1556

**Published:** 2015-07-15

**Authors:** Louise B Firth, Nova Mieszkowska, Lisa M Grant, Laura E Bush, Andrew J Davies, Matthew T Frost, Paula S Moschella, Michael T Burrows, Paul N Cunningham, Stephen R Dye, Stephen J Hawkins

**Affiliations:** 1 School of Geography, Earth and Environmental Science, Plymouth UniversityDrake Circus, Plymouth, PL4 8AA, UK; 2 Ryan Institute, National University of IrelandGalway, Ireland; 3 School of Ocean Sciences, Bangor UniversityMenai Bridge, Anglesey, LL59 5AB, UK; 4 The Laboratory, Marine Biological Association of the United KingdomCitadel Hill, Plymouth, PL1 2PB, UK; 5 CIESM – The Mediterranean Science Committee16 bd de Suisse, MC, 98000, Monaco; 6 Department of Ecology, Scottish Association for Marine Science, Dunstaffnage Marine LaboratoryOban, Argyll, PA37 1QA, UK; 7 Manchester Institute of Innovation Research, University of ManchesterManchester, M13 9PL, UK; 8 Former Department of Zoology, University of ManchesterManchester, M13 9PL, UK; 9 Marine Climate Change CentreCefas, Lowestoft, NR33 0HT, UK; 10 Centre for Ocean and Atmospheric Sciences, School of Environmental Sciences, University of East AngliaNorwich, UK; 11 Ocean and Earth Science, National Oceanography Centre Southampton, University of SouthamptonSouthampton, SO14 3ZH, UK

**Keywords:** Biogenic habitat, climate change, coastal defense structure, cold wave, extreme weather event, larval supply, monitoring, *Sabellaria alveolata*

## Abstract

Biogenic reefs are important for habitat provision and coastal protection. Long-term datasets on the distribution and abundance of *Sabellaria alveolata* (L.) are available from Britain. The aim of this study was to combine historical records and contemporary data to (1) describe spatiotemporal variation in winter temperatures, (2) document short-term and long-term changes in the distribution and abundance of *S. alveolata* and discuss these changes in relation to extreme weather events and recent warming, and (3) assess the potential for artificial coastal defense structures to function as habitat for *S. alveolata*. A semi-quantitative abundance scale (ACFOR) was used to compare broadscale, long-term and interannual abundance of *S. alveolata* near its range edge in NW Britain. *S. alveolata* disappeared from the North Wales and Wirral coastlines where it had been abundant prior to the cold winter of 1962/1963. Population declines were also observed following the recent cold winters of 2009/2010 and 2010/2011. Extensive surveys in 2004 and 2012 revealed that *S. alveolata* had recolonized locations from which it had previously disappeared. Furthermore, it had increased in abundance at many locations, possibly in response to recent warming. *S. alveolata* was recorded on the majority of artificial coastal defense structures surveyed, suggesting that the proliferation of artificial coastal defense structures along this stretch of coastline may have enabled *S. alveolata* to spread across stretches of unsuitable natural habitat. Long-term and broadscale contextual monitoring is essential for monitoring responses of organisms to climate change. Historical data and gray literature can be invaluable sources of information. Our results support the theory that Lusitanian species are responding positively to climate warming but also that short-term extreme weather events can have potentially devastating widespread and lasting effects on organisms. Furthermore, the proliferation of coastal defense structures has implications for phylogeography, population genetics, and connectivity of coastal populations.

## Introduction

**Figure 1:**
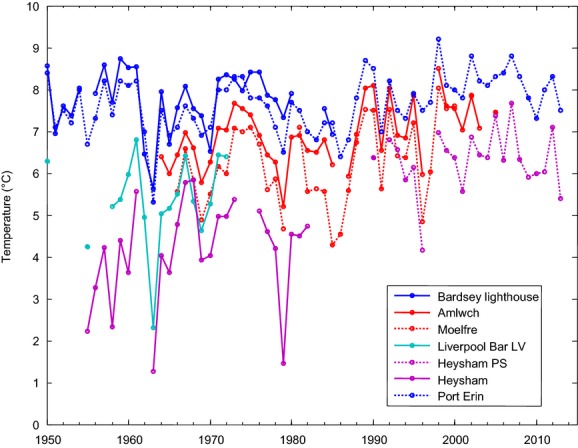
Average maritime winter (January–February–March) seawater temperature at seven sites around the eastern Irish Sea (locations as in Figure2) 1950–2013. [Data available through the Coastal Temperature Network maintained by Cefas, Lowestoft – [Data originators: Port Erin – The Isle of Man Government Laboratory (Department of Environment, Food & Agriculture) & University of Liverpool (Joyce, 2006). Bardsey Lighthouse – UK Met Office & University of Liverpool (Jones and Jeffs 1991); Amlwch – The Associated Octel Co. Ltd. (Joyce, 2006); Moelfre – Cefas (Joyce, 2006); Liverpool Bar LV – Cefas & University of Liverpool (Jones and Jeffs 1991); Heysham – NW and North Wales Sea Fisheries Committee (Jones and Jeffs 1991); Heysham PS – British Energy Generation UK Ltd., EDF Energy plc (Joyce, 2006)].

Global sea surface temperatures have been gradually rising since records began in 1861 (IPCC 2013). European seas are experiencing warming at twice the global average rate since 1960 (Burrows et al. 2011). The general warming trend has been punctuated by periods of cooling and warming (Fig.1) with recent extreme climatic events placing the last few years among the most extreme weather years on record (Francis and Vavrus 2012). At a regional scale, the unsmooth progression of warming is particularly evident in the Irish Sea. The Isle of Man, the warming trend in sea surface temperature for the last century (1904–2012) was 0.08°C decade^−1^ (Dye et al. 2013), whilst over 20-year period sea surface temperature (SST) here has warmed at a maximum rate of 0.7°C decade^−1^ (1985–2004) and cooled at a maximum rate of −0.3°C decade^−1^ (1968–1987) (Holt et al. 2012). Rising and stormier seas are leading to more severe coastal flooding and erosion, which is predicted to worsen over the next few decades (Wang et al. 2010). Hence, coastal habitats are subject to increasing environmental pressure from pervasive climate change interacting with other human impacts at regional and local scales (Hawkins et al. 2013a,b; Mieszkowska et al. 2014; Smale and Vance 2015).

In response to the threat of coastal erosion, artificial coastal defense structures are proliferating, particularly in urban areas (Chapman and Underwood 2011; Firth and Hawkins 2011; Firth et al. 2014). These structures have the potential to provide habitat for a range of marine organisms (Moschella et al. 2005; Firth et al. 2013a, 2015; Evans et al. 2015) including threatened canopy-forming algae (Perkol-Finkel et al. 2012), exploited molluscs (Devescovi and Iveša 2008; Martins et al. 2010), and non-native species (Mineur et al. 2012; Bracewell et al. 2013). In some situations, they may enable rocky shore species to colonize stretches of sedimentary coastline, thus potentially acting as “stepping-stones” to the spread of both native and non-native species (Johannesson and Warmoes 1990; Moschella et al. 2005; Mineur et al. 2012; Firth et al. 2013b; Airoldi et al. 2015).

Extreme environmental conditions have the potential to have significant and lasting effects on intertidal and shallow subtidal marine communities (Firth and Williams 2009; Firth et al. 2011; Wethey et al. 2011; Smale and Wernberg 2013; Smale and Vance 2015). Organisms living in the intertidal zone are of marine origin but are exposed to aerial conditions daily during low tide. This vulnerability to aerial conditions elicits strong responses in intertidal organisms that can result in changes in distribution, structure, and functioning (Helmuth et al. 2006; Simkanin et al. 2005; Hawkins et al. 2009, 2013a; Mieszkowska et al. 2013). For example, the extremely cold winter of 1962/1963 had a dramatic effect on marine organisms in Britain with widespread population decreases and localized extinctions (Crisp 1964).

The honeycomb worm *Sabellaria alveolata* Lamarck, 1812 is a Lusitanian warm-water species distributed from Morocco to southwest Scotland (Gruet 1986). This sedentary polychaete constructs tubes on low- to mid-shore hard substrata in semi-exposed and exposed locations and can form bioconstructions ranging from small patches, hummocks, and veneers to large reefs (Wilson 1971; Dubois et al. 2002, 2006). Artificial coastal defense structures are often built on sandy substrate in high-energy areas with plentiful sediment supply, thus representing potentially suitable habitat for *S. alveolata* (Frost et al. 2004). Reef-forming *S. alveolata* has the potential to provide important coastal protection (Naylor and Viles 2000) and biogenic habitat for a wide range of other species (Dubois et al. 2002; Cole and Chapman 2007).

Cunningham et al. (1984) produced the most extensive review of the distribution of *S. alveolata* in Britain. This report used historical data from the literature and new data from shore surveys and reports via correspondence with other researchers (including D. P. Wilson). As a result of this exercise, changes in the extent of *S. alveolata* distribution over a period of approximately 100 years were documented. Subsequently, Frost et al. (2004) carried out a broadscale survey of the distribution and abundance of *S. alveolata* near its northern range limit in Britain (from Anglesey, North Wales to Cumbria) and also used the extensive MarClim dataset (www.marclim.co.uk) to assess the UK distribution of *S. alveolata*.

Our aim was to combine “historical” (1919–1984, from Cunningham et al. 1984 and unpublished notebooks) and contemporary data (2002–2013, from Frost et al. 2004, MarClim dataset and surveys) to map past and present distributions of *S. alveolata* in relation to regional temperature variability. Specifically, we aimed to (1) describe spatiotemporal variation in mean winter temperatures across the region, (2) document short-term and long-term changes in the distribution and abundance of *S. alveolata* and discuss these changes in relation to extreme weather events and recent warming, and (3) assess the potential for artificial coastal defense structures to function as habitat for *S. alveolata*.

## Materials and Methods

### Spatiotemporal variation in temperature within the study region

Seven coastal and near-shore temperature stations for the region were used to examine spatiotemporal variation (Fig.1). The data were downloaded from the Coastal Temperature Network (Cefas, Lowestoft) at monthly resolution for the period 1950–2013 and averaged over January to March to give maritime winter means. The stations were selected to cover the entire study region from south (Bardsey) to north (Heysham). Only the station at Port Erin (maintained by Isle of Man Government Laboratory, see Kennington (2012)) ran continuously for the full period. Bardsey covered the first 30 years. Two stations on Anglesey (Amlwch and Moelfre) began after the cold winter 1962/1963 and were run until the early-to-mid 2000s. Data from the Liverpool Bar Light Vessel were collected until the early 1970s, whilst data from Heysham were collected from the 1950s and early 1980s and then from measurements at the local power station after 1990.

### *Sabellaria alveolata* sampling designs

**Figure 2:**
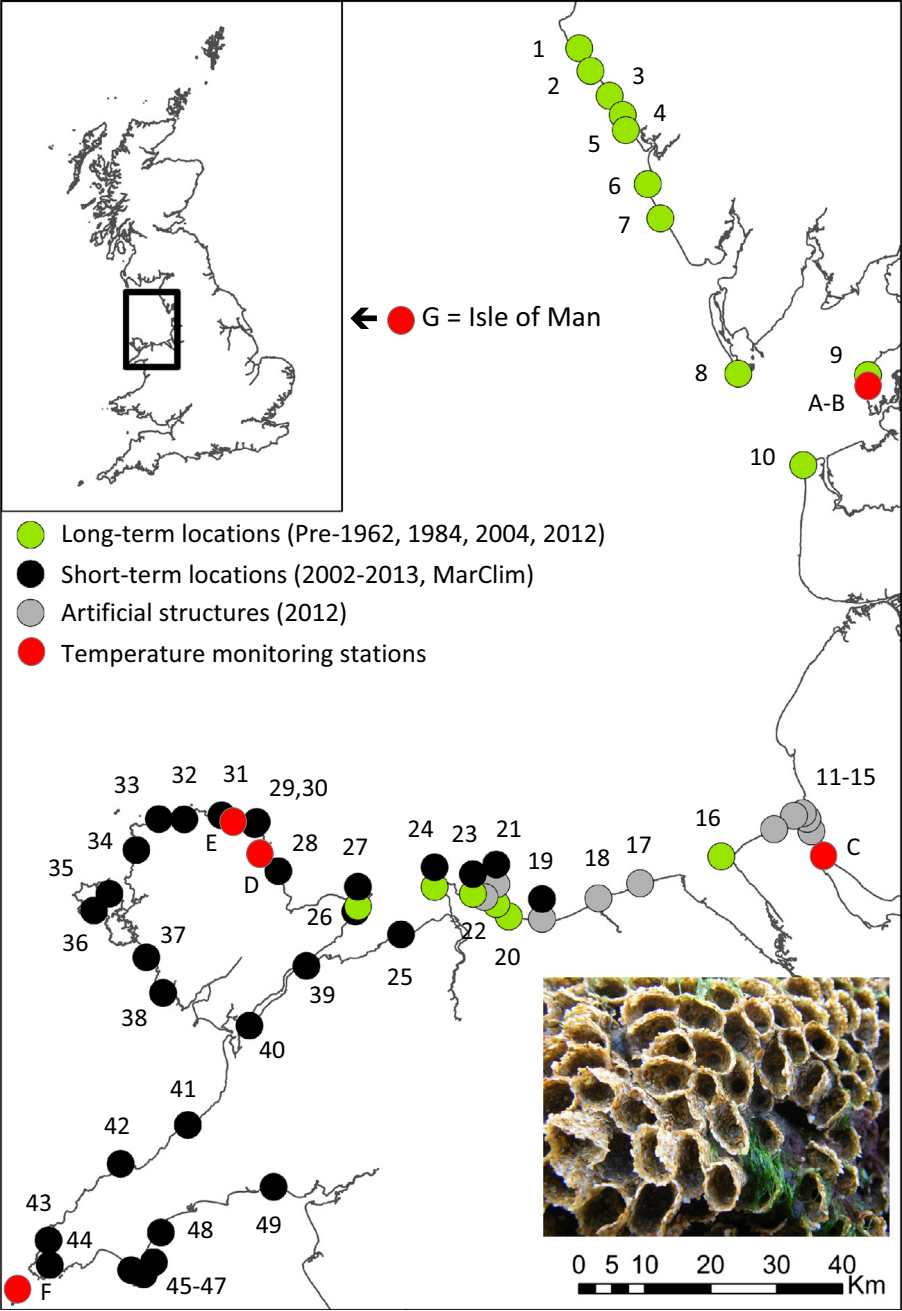
Map of survey locations in northwest England and Wales. Green dots represent long-term locations that were sampled pre-1962, 1984, 2004, 2012; Black dots represent MarClim locations that were surveyed in the short term (2002–2013); gray dots represent locations with artificial coastal defense structures that were surveyed in 2012 and red dots represent locations where sea surface temperature measurements were recorded. Note that some locations fall into more than one category (see Tables4 for explanation of survey location numbers). A = Heysham; B = Heysham Power Station; C = Liverpool Bar Light Vessel; D = Moelfre; E = Amlwch; F = Bardsey Lighthouse; and G = Port Erin (Isle of Man). Inset image shows healthy *S. alveolata* with obvious “porches” indicating live individuals.

A total of 49 locations were surveyed between St. Bees, Cumbria, and Criccieth, North Wales (Fig.2). Subsets of these locations were compared to address specific questions and are discussed in the relevant sections below.

#### Short-term and long-term change on natural shores

Short-term (2002–2013) variability and long-term (1919–2013) variability in the distribution and abundance (Table1) of *S. alveolata* were compared between 29 and 16 locations, respectively. Locations for short-term comparisons (Llanddulas to Criccieth, North Wales, Table2) were selected if they were deemed potentially suitable habitats for *S. alveolata* and if sufficient surveys were available from the MarClim intertidal biodiversity dataset (www.marclim.co.uk). Locations for long-term comparisons were selected based on those surveyed by Cunningham et al. (1984) and Frost et al. (2004) (St. Bees, Cumbria to Penmon, Anglesey, Table3). Locations for long-term comparisons were divided into four periods based on previous surveys and available data: (1) pre-1962 (before the cold winter 1962/1963), (2) 1984 (following a cold period after the cold winter 1962/1963), (3) 2004 (following a period of climate warming), and (4) 2012 (following a period of continued warming).

**Table 1:** Scale of abundance used to record *Sabellaria alveolata*. Adapted from Cunningham et al. (1984).

Abundance	Description
N	Not seen: Absent
R	Rare: <10 found in 30 min search
O	Occasional: Scattered individuals, no patches
F	Frequent: Many scattered individuals and small patches
C	Common: Large sheets or patches at some shore levels (not forming large hummocks)
A	Abundant: large colonies, forming hummocks over 1 ft across, more than 20% cover overall at shore levels of peak abundance

**Table 2:**
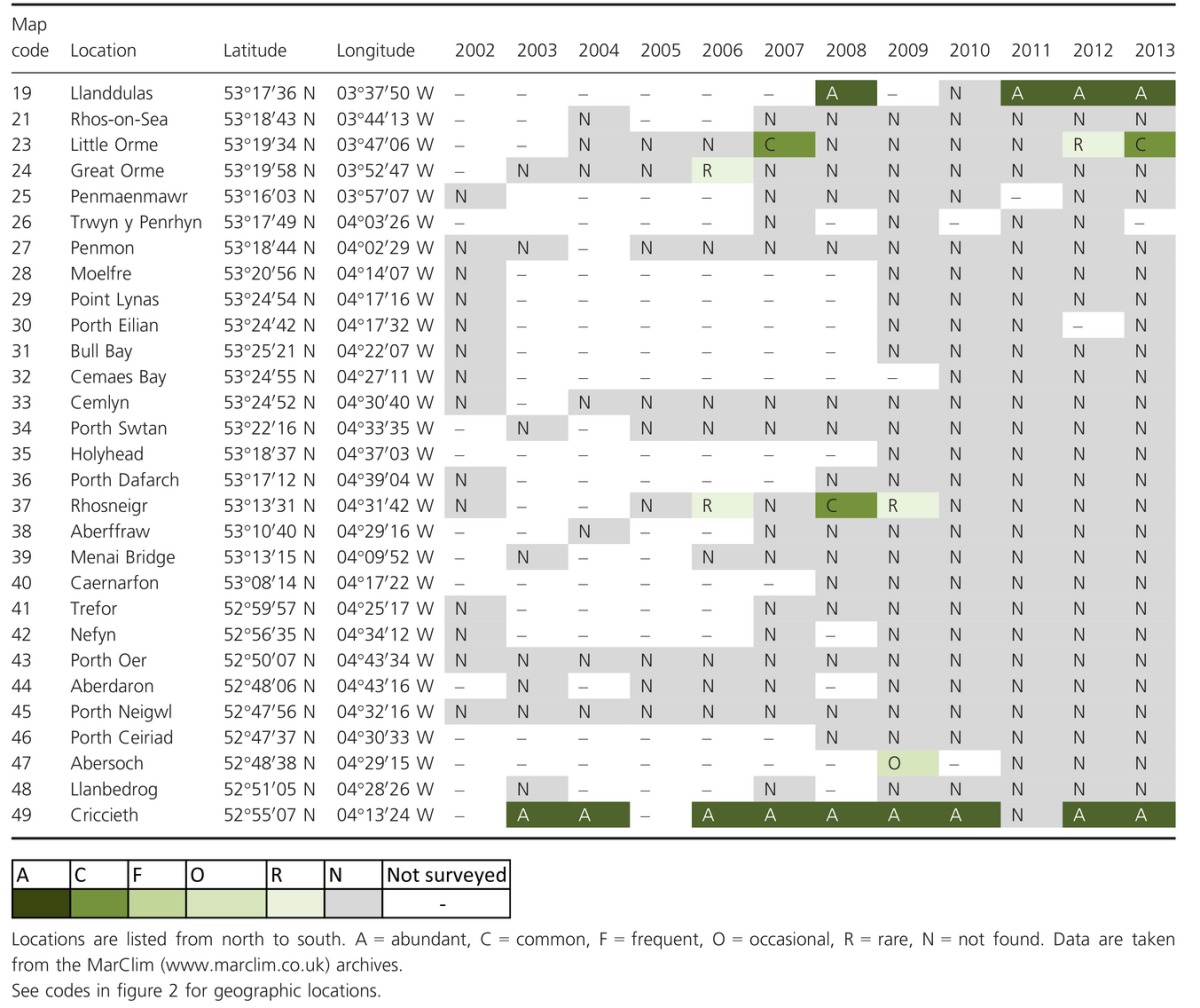
Short-term comparisons. Interannual variation in maximum ACFOR abundance of *Sabellaria alveolata* at 29 locations across Wales.

**Table 3:**
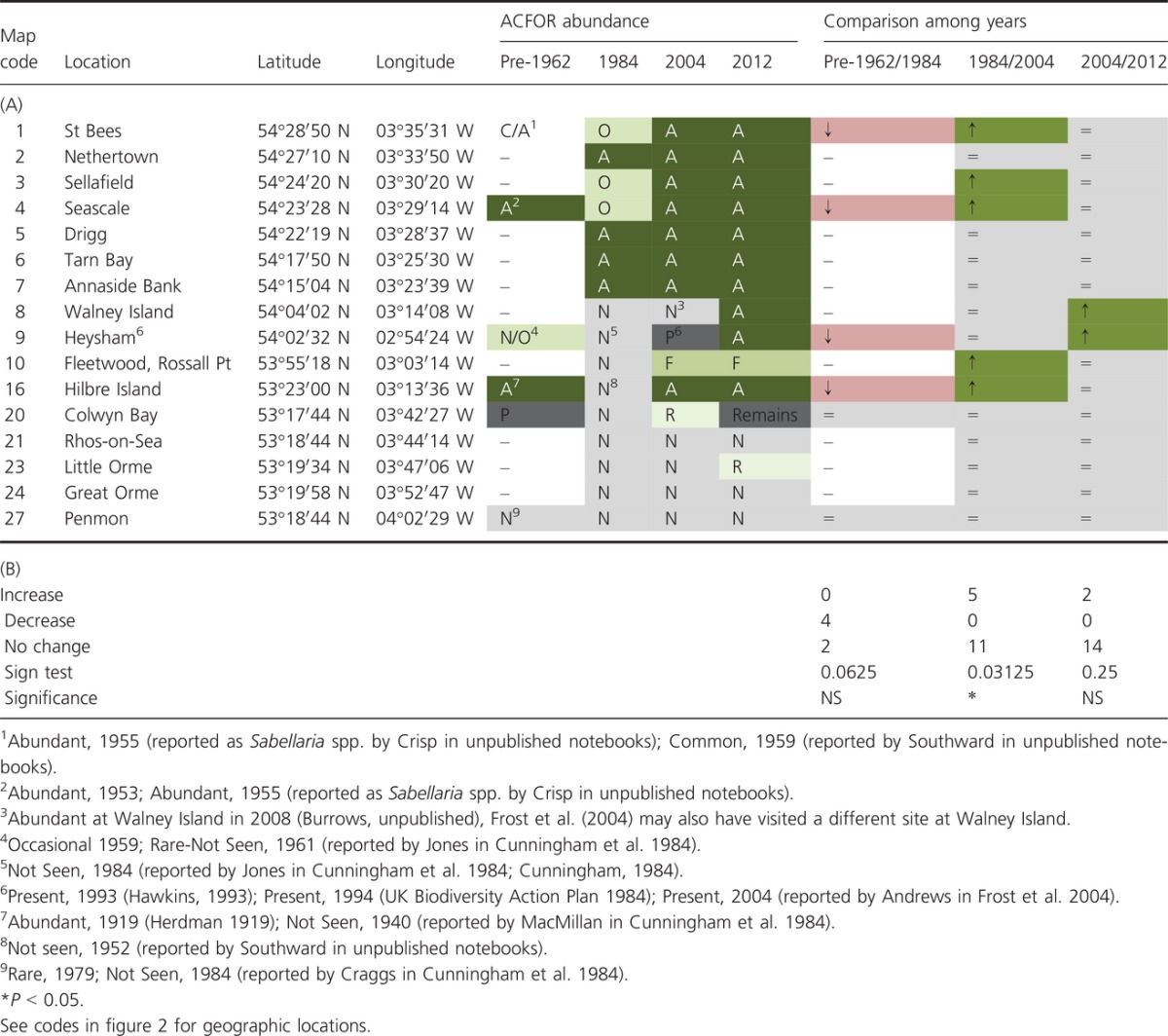
Long-term comparisons. (A) Historical and contemporary data on *Sabellaria alveolata* maximum ACFOR abundance in natural habitats in northwest England and Wales. Locations are listed form north to south. A = abundant, C = common, F = frequent, O = occasional, R = rare, N = not found, P = recorded as present but abundance not known. The relative changes in abundance (based on a two-step category change) to the previous survey(s) are shown. “↑” denotes increase, “↓” denotes decrease and “=” denotes no change. “Remains” = tubes of dead individuals. Pre-1962 data taken from Cunningham et al. (1984) and notebooks of Denis Crisp and Alan Southward. 1984 data taken from Cunningham et al. (1984). 2004 data taken from Frost et al. (2004). (B) Results of binomial sign tests comparing the number of locations exhibiting increased, decreased (based on a two-step category change), and no change in abundances among sampling periods.

The number of locations where *S. alveolata* exhibited a change in abundance (increase or decrease) was compared using one-tailed sign tests for each of the paired periods (i.e., pre-1962/1984, 1984/2004, and 2004/2012) with the *a priori* assumptions that this warm-water species would decrease in abundance following the cold winter of 1962/1963 and that it would increase in abundance over recent warming periods. Due to the subjective nature of the ACFOR abundance scale (see Survey Techniques below, Table1), we only assigned a change in abundance to locations that exhibited a two-category change (e.g., frequent to abundant) rather than a one-category change (e.g., common to abundant). This enabled greater confidence in identifying changes in abundance across sampling periods.

#### Artificial coastal defense structures

In order to assess the potential for artificial coastal defense structures to provide habitat for *S. alveolata*, a total of 10 artificial coastal defense structures were surveyed between the Mersey Estuary and Penrhyn Bay, North Wales, in July 2012 (Table4). Natural locations supporting *S. alveolata* occur between these defense structures at Hilbre, Llanddulas, and Colwyn; westwards at Little Orme and northwards at Fleetwood (Fig.2).

**Table 4:**
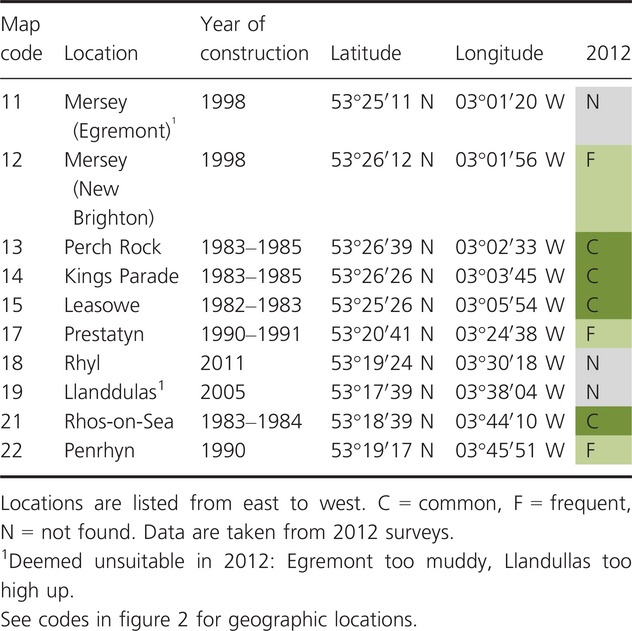
Artificial coastal defense structures. Comparison of maximum ACFOR abundance of *Sabellaria alveolata* at 10 artificial coastal defense structures across North Wales and the Wirral.

### Survey techniques

*Sabellaria alveolata* was recorded using the modified semi-quantitative ACFOR abundance scale (abundant, common, frequent, occasional, rare, Table1). Following a 30-min search, a score was assigned to the zone of maximum abundance. This cost-effective and rapid survey technique was employed firstly, to ensure comparability among surveys (Cunningham et al. 1984; Frost et al. 2004; MarClim protocols) and secondly to enable broad coverage over short timescales. Hawkins surveyed on both the 1984 and 2004 surveys. He in turn coached Firth and Mieszkowska to ensure consistency across surveys and was involved in North Wales surveys from 2007 to 2010.

As previous studies used grid references rather than global positioning system (GPS) coordinates to identify locations, it was difficult to know exactly where these historical locations were situated. Google Earth and Ordnance Survey maps were used to aid in the identification of areas of suitable habitat.

## Results

### Spatiotemporal variation in temperature within the study region

Trends in sea surface temperature (SST) vary depending on the time period for which they are assessed (e.g., Holt et al. 2012). Port Erin was the only station sampled consistently from 1950 to 2013. Over this period, annual mean temperatures measured at Port Erin warmed at an average rate of 0.13°C decade^−1^. For multidecadal trends in winter temperatures taken within the study area, we can compare the 40-year period 1966–2005 at three stations. The coastal seawater temperature on the east coast of Anglesey at Amlwch and Moelfre rose by 0.25°C decade^−1^ and 0.37°C decade^−1^, respectively, and by 0.21°C decade^−1^ at Port Erin. For the 20-year period 1968–1987, identified at Port Erin as a cooling period by Holt et al. (2012), winter SST also cooled at Bardsey Lighthouse (−0.14°C decade^−1^), Amlwch (−0.26°C decade^−1^), and Moelfre (−0.50°C decade^−1^).

The data from Port Erin also clearly illustrate that 1962/1963 was an extremely cold winter with a mean winter temperature of 5.3°C observed between January and March 1963. Three other locations were operational during this period with Liverpool Bar and Heysham reporting even lower temperatures of 2.3°C and 1.3°C, respectively (Fig.1). Figure1 also demonstrates that particularly cold winters were evident across the study area and across the eastern Irish Sea in 1978/1979, 1985/1986, and 1986/1987. More recently, the winters of 1995/1996 and 2009/2010 were cold relative to the generally warmer conditions that have persisted since about 1987. The 1978/1979 winter also emerged as a cold winter at some locations, but temperatures varied greatly across the region; the coldest temperatures at Heysham in the north (1.5°C) compared to the warmest at Bardsey in the south (7.3°C).

From south to north, the sea water temperature is seen to generally decrease, with Bardsey consistently exhibiting the warmest temperature and Heysham consistently exhibiting the coldest temperature (Fig.1). For the 5-year period when all locations were monitored coincidently (1968–1973), the range in winter temperature is almost 3°C across the region varying from 4.7°C at Heysham to 7.6°C at Bardsey. The difference in temperature between locations is high compared to the long-term trends (tenths of a degree per decade) and the interannual variability at a site (interannual winter standard deviations: 0.7–1.2°C). All of the stations were strongly positively correlated with Port Erin (*P* < 0.05); for the two Heysham series (Heysham *r*^2^ = 0.48, Heysham Power Station *r*^2^ = 0.44), this relationship is weaker than for the other series (*r*^2^ > 0.72).

### Short-term change in distribution on natural shores

*Sabellaria alveolata* was consistently recorded as absent at 23 of the 29 locations surveyed (Table2). Of the six locations where it was recorded, two appeared to support persistent populations of *S. alveolata* (Llanddulas and Criccieth), whilst the remaining four supported transient populations (Little Orme, Great Orme, Rhosneigr, and Abersoch). Four locations exhibited a population decrease or local extinction following the cold winters of 2009/2010 and 2010/2011 (Llanddulas, Rhosneigr, Abersoch, and Criccieth).

### Long-term change in distribution on natural shores

**Figure 3:**
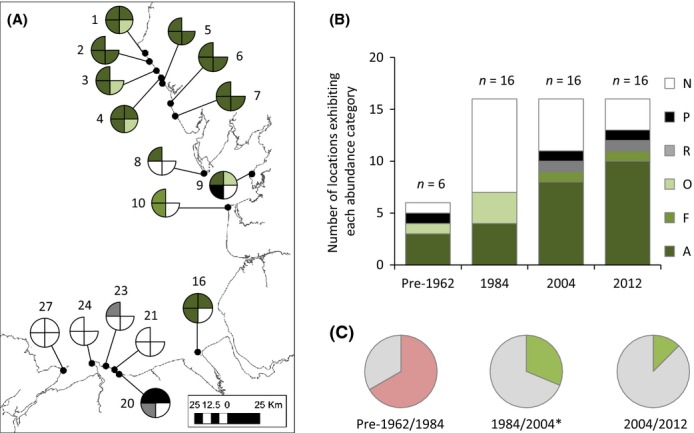
(A) Spatiotemporal comparison of distribution and abundance of *Sabellaria alveolata* across long-term survey locations (St. Bees Cumbria to Penmon, Anglesey). See Table3 for explanation of survey location numbers. Each quadrant of the pie chart represents a survey: top right = pre-1962 (from Cunningham et al. 1984 and Crisp and Southward unpublished notebooks); bottom right = 1984 (from Cunningham et al. 1984); bottom left = 2004 (from Frost et al. 2004); top right = 2012 (collected by authors). No data available for some locations in 1962 (denoted by empty quadrant). See legend for color coding. A = abundant; C = common; F = frequent; O = occasional; R = rare; P = present; N = not seen. (B) Comparison of the relative proportions of each abundance category among sampling periods: Six locations were sampled in pre-1962 and 16 locations were sampled in 1984, 2004, and 2012. (C) Pie charts illustrating the comparison of the proportion of locations exhibiting no change (gray) and decreased abundances (pink) and increased abundances (green) among paired sampling periods: pre-1962/1984; 1984/2004, and 2004/2012. *P < 0.05.

Despite the paucity of data available prior to 1962, there are reliable records from St. Bees, Heysham, Seascale, Colwyn, and Hilbre (Table3, Fig.3). In 1984, *S. alveolata* was completely absent from Anglesey to Barrow-in-Furness (Cunningham et al. 1984), exhibiting decreases at four (St. Bees, Seascale, Heysham, and Hilbre) of the six locations in 1984 (Table3). A significant increase was observed in 2004 (Table3), where *S. alveolata* had recolonized four locations where it had previously been reported as absent (Heysham, Fleetwood, Hilbre, and Colwyn) and had increased at a further three locations (St. Bees, Sellafield, and Seascale; Fig.3, Table3). A further increase was observed at two locations in 2012 (Heysham and Walney Island; Fig.3, Table3).

### Artificial coastal defense structures

**Figure 4:**
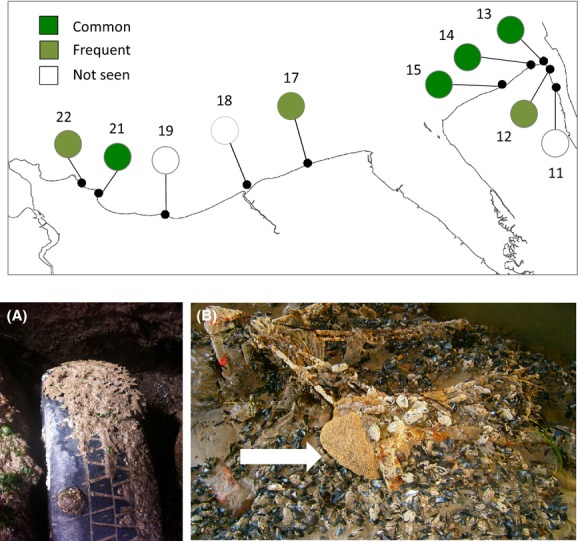
Distribution and abundance of *Sabellaria alveolata* on artificial coastal defense structures along the North Wales and Wirral coasts. See legend for color coding. See Table4 for explanation of survey location numbers. Photographs illustrating *Sabellaria alveolata* colonizing novel hard substrata in the study area. (A) A small patch colonizing a half-buried car tire at Penrhyn Bay, North Wales (Photo: Laura Bush). Note that *S. alveolata* has the potential to colonize artificial hard substrata. (B) A small colony (foreground) on a shopping trolley on the Wirral (Photo: John Lamb).

*Sabellaria alveolata* was recorded on 7 of the 10 artificial coastal defense structures surveyed (Fig.4, Table4). It was usually found as veneers between the blocks at the base of the structures. Of the three structures that it was absent on, two of these were considered to be unsuitable due to being positioned too high up on the shore (Llanddulas), or the presence of muddy sediments around the base of the structure (Mersey Egremont). Interestingly *S. alveolata* was found colonizing a discarded tire and a half-buried shopping trolley (Fig.4), demonstrating that *S. alveolata* may be opportunistic, even colonizing what appears to be undesirable artificial substrata.

## Discussion

Using a combination of the best available historical and contemporary data (spanning ∼100 years), we provide an account of broadscale and long-term changes in the distribution and abundance of an important biogenic habitat near its range edge in Britain. Without experimental manipulation, it is impossible to assign any causal effects to these changes with any real certainty, but we discuss the findings in relation to prolonged cold winters, short cold waves, recent warming, and the proliferation of artificial coastal defense structures in the study area.

There has been an overall positive warming trend in SST in the Irish Sea whether measured over the last century or the last 60 years (Fig.1). This overall warming trend has been punctuated by cooling periods (1968–1987) and by extremely cold winters such as 1962/1963 and 1979 (Holt et al. 2012; Dye et al., 2013). In recent years, the warming trajectory has levelled off somewhat, with short cold events experienced in 2009/2010 and 2010/2011. Despite the apparent cooling trajectory in the latter observations, 2014 was recognized as the warmest year in Britain (and globally) since records began (Scientific American, 2014; UK Met Office, 2014).

### Fluctuation in abundance due to climate warming and extreme weather

#### Decreases in abundance in response to extreme weather events

*Sabellaria alveolata* disappeared from the North Wales and Wirral coastlines following the cold winter 1962/1963 and was not recorded again until 40 years later in 2004 (Fig.3, Cunningham et al. 1984; Frost et al. 2004; Mieszkowska et al. 2006). Crisp (1964) reported mass mortality of *S. alveolata* immediately after the cold winter of 1962/1963 at many locations in both North and South Wales. At Criccieth, heavy losses (up to 95%) were recorded with reefs above the LWNT level being “completely destroyed.” Despite this devastation, immediate signs of recovery were reported with survivors building new reefs at Criccieth by April 1963 (Crisp 1964). The reefs at Criccieth were badly affected further by the cold winter of 1984 (Gubbay 1988). It is assumed that mortality along the North Wales coastline was complete (100%, with no survivors to begin rebuilding) and that extremely cold air temperatures (in addition to cold water temperature) were a major driver of this mortality event (see Firth et al. 2011).

The cold winter of 1962/1963 had a devastating effect on marine life in Britain (see Crisp 1964 for review). The Lusitanian intertidal gastropod *Phorcus lineatus* exhibited a similar recolonization pattern in the region to *S. alveolata* (see Mieszkowska et al. 2007). In the 1950s, the northern limit of distribution of *P. lineatus* in Britain was Point Lynas, Anglesey (Crisp and Knight-Jones 1954). Populations were eradicated or cut back in the severely cold winter of 1962/1963 (Crisp 1964) to the south of the Lleyn Peninsula in North Wales, and recolonization of sites was minimal throughout the 1980s and 1990s, with only isolated individuals being found at Rhosneigr, Anglesey, in the mid-1980s and in 1992 (S. J. Hawkins, pers. obs.). Following the intervening warming period (Fig.1), the range limit of *P. lineatus* extended northwards to Anglesey in 2002 (Mieszkowska et al. 2007) and eastwards to Llandudno, North Wales, in 2012 (MarClim surveys, Hawkins et al. 2013a).

Although not experimentally tested, the data reported here (Fig.3, Table3) provide evidence in support of the assertion that the cold winter 1962/1963 led to total mortality and long-term absence of *S. alveolata* along the North Wales and Wirral coast (Frost et al. 2004). Subsequent recolonization was probably inhibited by cooler conditions in much of the 1960s to 1980s (Fig.1). Furthermore, recolonization may have been inhibited by limited larval supply. The recovering reefs at Criccieth, North Wales (Crisp 1964), represented a potential source population to the south. Due to lack of data, it is unknown whether the Cumbria populations were wiped out by the cold winter, but it is assumed that mass mortality occurred in this region also, due to the exceptionally low temperatures recorded at Heysham (Fig.1). However, if there were survivors that began rebuilding, these locations may have represented source populations to the north. Even if there were potential source populations to the south and north, it is highly unlikely that larvae would have reached the North Wales and Wirral coasts due to local hydrodynamics in Liverpool Bay (Robins et al. 2013).

The recent cold winters of 2009/2010 and 2010/2011 appear to have had a similar negative effect on *S. alveolata* in the region, albeit not on the same spatial or temporal scales (Table2). Although the recent cold winters were nowhere near as harsh as 1962/1963, they did have a significant effect on marine life, particularly organisms inhabiting the intertidal zone. For example, Wethey et al. (2011) describe differential responses of species along the French and Iberian coasts with Boreal species exhibiting positive responses (i.e., recruitment success and range expansion) and Lusitanian species exhibiting negative responses (recruitment failure) to the cold conditions. Similarly, in the United States, the cold winter of 2007 caused mass mortality of intertidal populations of the invasive mussel *Perna viridis* in Florida but populations recovered, possibly due to the survival of subtidal populations (Firth et al. 2011).

#### Increases in abundance in response to recent warming

Overall, *S. alveolata* increased in abundance at 7 of the 16 locations (Fig.3, Table3) surveyed during a warming period between the mid-1980s and the 2000s (Fig.3). In addition to increasing at three locations where it had previously been recorded in 1984, *S. alveolata* was recorded for the first time at a further six locations in the 2000s, suggesting recruitment success at locations where it was previously absent. It is difficult to say whether this increase and new colonization is a direct result of warming temperatures, but the pattern is certainly in line with observations for a range of other intertidal and fish species in the region (e.g., Mieszkowska et al. 2006, 2013; Hiddink and ter Hofstede 2008; Firth et al. 2009; Hawkins et al. 2013a; Smale et al. 2013).

#### Artificial coastal defense structures

*Sabellaria alveolata* was found on the majority of artificial coastal defense structures that were surveyed. In the absence of the actual structures, locations that comprise natural hard rocky substrata may indeed represent suitable habitat for *S. alveolata*. For example, *S. alveolata* is present on the natural boulder field surrounding the groin at Penrhyn Bay but not at Rhos-on-Sea, which is perhaps surprising as it appears to be suitable habitat and *S. alveolata* is present on the groin. Frost et al. (2004) suggested that artificial coastal defense structures represent potential habitat for *S. alveolata*, as they typically comprise hard substrate within areas of sand and particle supply in locations that have moderate to heavy wave action. Many new structures have been built along the North Wales and Wirral coastlines since the 1980s, with some very large schemes being undertaken in recent years (e.g., Colwyn and Rhyl). Recent work has revealed that *S. alveolata* had colonized newly built artificial coastal defense structures within 6 months of construction at Borth and Tywyn in Cardigan Bay, Wales (Firth et al. 2013a; Evans et al. 2015). Furthermore, Pearce et al. (2014) observed increases in abundance of the conspecific *Sabellaria spinulosa* following construction of a large-scale wind farm site off the Kent coast, UK. It was concluded that the wind farm may be providing some protection from damage caused by fishing gears to this important biogenic habitat.

All of the coastal defense structures surveyed during this study were within the natural distribution range of *S. alveolata*. If defense structures are located adjacent to a stretch of unsuitable soft sediment habitat, or just beyond the range edge of a rocky shore species, the structures may function as “stepping-stones” to dispersal between disparate populations and/or facilitate range extensions (Frost et al. 2004; Mineur et al. 2012; Firth et al. 2013b). Examples of range extensions that were potentially facilitated by artificial structures exist for gastropods on the south coast of England (e.g., *Gibbula umbilicalis, Melaraphe neritoides,* Moschella et al. 2005) and along the Belgian coastline (e.g., *Littorina saxatilis*, Johannesson and Warmoes 1990).

#### Monitoring challenges and opportunities

When considered in isolation, Cunningham et al. (1984) provided a snapshot of the distribution and abundance of *S. alveolata* on a UK-wide scale. Frost et al. (2004) built on this valuable baseline dataset and provided a spatiotemporal comparison, focussing near the northern range edge. The 2012 survey built on this again providing a short-term comparison with Frost et al. (2004).

In order to detect the impacts of future extreme weather events and climate change, we recommend that monitoring of fixed areas be carried out over short (annual), medium (5–10 years), and long (20–30 years) timescales. The current known northern distribution limit of *S. alveolata* is Solway Firth, SW Scotland (Bush, unpublished data). It is anticipated that this range limit will extend northwards in response to climate warming, although coastal topography could present a hydrographic barrier in its spread northwards (Keith et al. 2011). We recommend that the ACFOR scale be used for broadscale monitoring and that the number of locations be increased. We also recommend that this be combined with quantitative survey techniques (transects, aerial mapping) at a range of spatial scales to gain a better understanding of densities from locations of particular interest. For example, range edges (SW Scotland) and hotspots (e.g., Criccieth, Llanddulas, and Annaside) should be targeted for quantitative monitoring in addition to any new artificial coastal structures.

The ACFOR scale does not take condition into account; therefore, dead and live reef can be given the same abundance category. Desroy et al. (2011) described a biological health index for *S. alveolata* to serve as an easy-to-use management tool for the identification and protection of threatened reefs. This method involves taking cores that can only be done on relatively large reef-forming populations, such as those in Mont Saint Michel Bay (Dubois et al. 2002, 2005, 2006), and therefore is not a tool that can be widely applied across its geographic distribution. Natural Resources Wales (NRW, formerly the Countryside Council for Wales) and the UK Environment Agency (EA) have both created condition descriptors for *S. alveolata* (see Boyes et al. 2008). For the purpose of long-term, broadscale (and cost-effective) sustained monitoring, a generic rapid assessment health/condition index (e.g., those proposed by NRW, EA) that can be used in conjunction with the ACFOR scale would provide a valuable metric combining both the abundance and condition of *S. alveolata* populations.

## Conclusions

This study presents data from 49 locations that were surveyed using the same sampling protocols during three different surveys spanning a warming period between 1984 and 2012. It describes the changes in distribution of an important ecosystem engineer in relation to short-term extreme weather events, recent warming and the construction of a network of artificial coastal defense structures near its northern range edge in Britain. We combined contemporary and historical datasets that were only previously available in reports (Cunningham et al. 1984; Frost et al. 2004). Without long-term and broadscale contextual monitoring, it is difficult to assess whether observed changes are a result of long-term climate change, shorter-term responses to weather, or other local impacts (Hawkins et al. 2013a,b; Mieszkowska et al. 2014). Gray literature and historical records are invaluable in such comparative studies.

We present new information regarding the temporal stability of this important habitat that has not previously been recorded in the published literature, and it is anticipated that this will be of significant use to those tasked with the management and protection of these habitats. Assessing variability in habitat distribution and abundance is also important at a time when the implementation of some marine policies (such as the establishment of marine reserves) assumes a degree of stability in the features being protected. There is a wealth of gray literature (reports and unpublished theses) and unpublished data (field notebooks) available that contain vital historical records, but can often be difficult to access. Furthermore, information is available through national databases. Examples from the UK include the National Biodiversity Network (NBN), the Marine Nature Conservation Committee (MNCR), and the Marine Life Information Network (MarLIN). The data contained in these sources provide the raw material for data mining and can be combined to produce broadscale and long-term datasets. In this current climate of cuts in state-funded science, historical and unpublished data represent a potentially invaluable resource for sustained monitoring (Hawkins et al. 2013a).
